# A novel mass spectrometry method to identify the serum monoclonal light chain component in systemic light chain amyloidosis

**DOI:** 10.1038/s41408-019-0180-1

**Published:** 2019-02-04

**Authors:** Faye A. Sharpley, Richa Manwani, Shameem Mahmood, Sajitha Sachchithanantham, Helen J. Lachmann, Julian D. Gillmore, Carol J. Whelan, Marianna Fontana, Philip N. Hawkins, Ashutosh D. Wechalekar

**Affiliations:** National Amyloidosis Centre, University College London, London, UK

AL amyloidosis is characterised by an underlying plasma cell clone producing structurally abnormal monoclonal free light chains (FLCs) which mis-fold and deposit as amyloid fibrils leading to progressive tissue damage. Detection and serial measurements of serum FLCs are critical in determining prognosis and in assessing response to treatment. All current immunoassays for quantifying amyloidgenic monoclonal FLCs also measure the normal polyclonal FLC background—a major limitation for a disease where even low-level monoclonal FLCs are crucially important. We describe a novel mass spectrometry (MS) method for accurate detection of *monoclonal* free light chains in patients with systemic AL amyloidosis.

MS is a technique used to sort a sample based on the ratio of mass to charge. MS has recently been explored in the assessment of FLCs both in the setting of AL amyloidosis and other plasma cell dyscrasias;^1^ the theory being that each monoclonal FLC is made of a unique amino acid sequence, with a unique molecular mass. Various different MS techniques exist. The clonotypic peptide MS approach relies on the digestion of serum immunoglobulins with trypsin prior to analysis by MS^2^. Although this approach is sensitive^3^, the technique relies on the initial identification of a peptide from the patient’s monoclonal protein (M protein)/FLC, which can then be serially monitored over time. An alternative approach is the monoclonal immunoglobulin rapid accurate molecular mass (miRAMM) technique which, rather than analysing tryptic peptides, utilises a reducing agent to dissociate the heavy and light chains allowing MS analysis of intact proteins. This allows both post-translational modification change to be observed^1^ and minor FLC sub-clones to be monitored^1^. The matrix-assisted laser desorption ionization time of flight mass spectrometry (MALDI-TOF or MASS-FIX) is a high throughput version of miRAMM^4^ which has been explored in a group of patients with plasma cell dyscrasia and has demonstrated comparable sensitivity to existing protein electrophoresis and serum FLC methods^1^.

Here we report on a novel and simple to use MALDI-TOF-MS method for monoclonal FLC detection (FLC-MS) in a small series of patients with systemic AL amyloidosis.

We included 17 serial patients with systemic AL amyloidosis, one patient with amyloid of uncertain type, and two MGUS (monoclonal gammopathy of undetermined significance) patients, all referred to the UK National Amyloidosis Centre (UK-NAC) (Table 1). Two of the 17 patients with AL amyloidosis were selected with samples at diagnosis and post-treatment when in complete remission (CR), but with known presence of minimal residual disease (MRD) on bone marrow. Sera samples from healthy donors (*n* = 17), with normal kappa and lambda ratios, were also analysed using sheep polyclonal antisera (Binding-Site, Birmingham, UK) with equivalent specificity for comparison (data not shown). A diagnosis of AL amyloidosis was confirmed by demonstration of characteristic birefringence under cross-polarized light with Congo-red staining on a tissue biopsy, and AL typing was confirmed by immunohistochemistry or by laser capture mass spectrometry. All patients had detailed baseline assessments of organ function including serum FLC measurements and imaging. Organ involvement and organ response was defined according to the international amyloidosis consensus criteria^5^.

**Table 1:** Baseline characteristics of amyloidosis patients, *n* = 18*

	*n* (%)	Median (range)
Organs involved:		2 (1–4)
*Cardiac*	14 (77)	
*Renal*	8 (44)	
*Autonomic and soft tissue*	3 (17)	
*Peripheral nerve*	1 (6)	
*Liver*	0 (0)	
NT-proBNP, ng/L		3761 (245–25348)
Troponin T, ng/L		35 (8–170)
Serum albumin, g/L		37 (19–45)
eGFR, mL/min		62 (10–100)
Amyloid type:		
*AL kappa*	3 (17)	
*AL lambda*	14 (78)	
*Uncertain*	1 (6)	
iFLC kappa, mg/L		78 (73–440)
iFLC lambda, mg/L		185 (44–1023)
dFLC, mg/L		118 (33–1015)
Monoclonal Intact Ig	14(77)	

*two *MGUS* patients not included in the table

*NT-proBNP* indicates N-terminal pro b-type natriuretic peptide, *eGF*R estimated glomerular filtration rate, *AL* light chain amyloidosis, *iFLC* involved free light chain, *dFLC* difference between involved and uninvolved free light chain, *Ig* immunoglobulin

Commercially available paramagnetic microparticles were covalently coated with polyclonal sheep antibodies monospecific for human kappa FLCs (anti-free κ) and lambda FLCs (anti-free λ) (Binding-Site, Birmingham, UK). The microparticles were incubated with patient sera, washed and treated with acetic acid (5% v/v), containing tris(2-carboxyethyl)phosphine (TCEP) (20 mM), in order to elute FLCs in monomeric form. Mass spectra were acquired on a Microflex LT/SH smart matrix-assisted laser desorption ionization time-of-flight mass spectrometer (MALDI-TOF-MS; Bruker, GmbH). Approval for analysis and publication was obtained from the NHS institutional review board, and written consent was obtained from all patients in accordance with the Declaration of Helsinki.

**Fig. 1:**
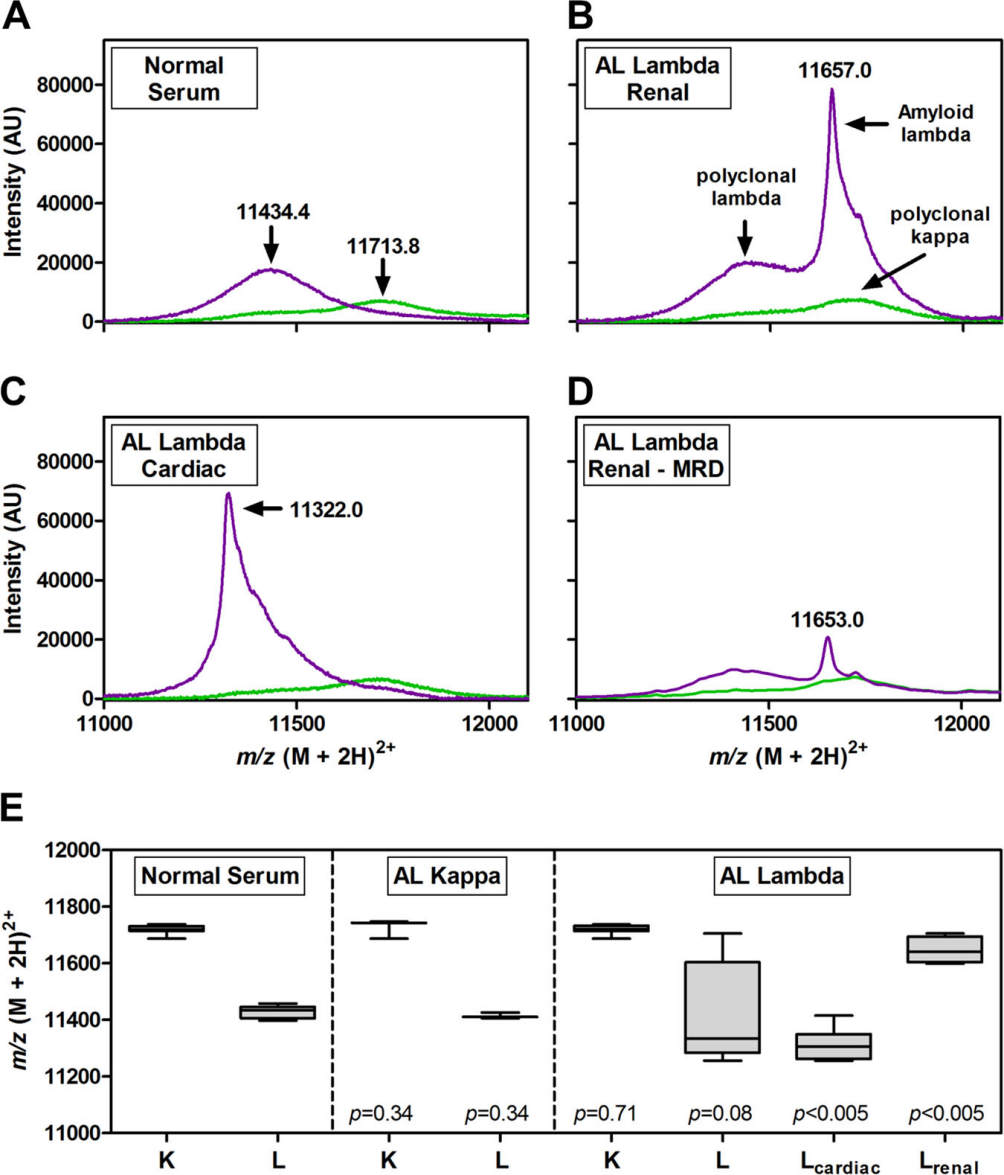
Mass spectra and m/z values for FLC in patients with AL amyloidosis. Examples of kappa and lambda FLC molecular mass of **a** a normal control; **b** a patient with lambda amyloidosis and renal involvement demonstrating a “heavy mass” monoclonal FLC; **c** a patient with lambda amyloidosis and cardiac involvement demonstrating a “light mass” monoclonal FLC; **d** a patient with lambda amyloidosis and renal involvement in haematological complete response (CR) after treatment, demonstrating a “heavy mass” monoclonal FLC; **e** comparative median m/z FLC values from control, kappa and lambda AL amyloidosis patients; lambda FLC values for patients with cardiac and renal involvement are presented. Statistical differences relative to controls were assessed by Mann-Whitney *U*-test: *p* < 0.05 indicates significance. Values for *m/z* represent the 2+ charged ions. Mass spectra for kappa FLCs are shown in green; for lambda FLC in purple

The baseline characteristics of patients are presented in Table 1. The FLC-MS assay confirmed normal polyclonal kappa and lambda FLC expression in the 17 controls. The FLC-MS assay correctly identified the presence and type of monoclonal FLC in 3/3 (100%) kappa and 14/14 (100%) lambda AL amyloidosis patients (Fig. 1a–c). The FLC-MS assay did not detect any monoclonal FLC in one patient with amyloid of uncertain type, where amyloid fibril type remained unclear by both immunohistochemistry and laser capture mass spectrometry, suggesting against a diagnosis of AL amyloidosis.

In two patients, FLC-MS identified presence of monoclonal lambda FLC with the same molecular mass (respectively) with paired samples at diagnosis and following achieving a serological CR post-treatment (Fig. 1d) (in both cases with normal FLC (lambda light chains < 20 mg/L in each case), and no monoclonal band in immunofixation in serum and urine). Both patients achieved an organ response to treatment. The first patient had renal involvement alone and achieved a renal response with a 61% reduction in proteinuria, but with persistent proteinuria of 2.1 g/day. The second patient had cardiac involvement and achieved a cardiac response with an 88% reduction, and normalisation, of NT-proBNP levels. Both patients had had a bone marrow examination with next generation sequencing (NGS) and next generation flow cytometry (NGF), respectively, showing persistent MRD in the bone marrow to the level of < 40 cells/10^6^.

In lambda AL amyloidosis patients with renal involvement, the monoclonal lambda FLC tended to display a “heavy” molecular mass (*m/z*^[2+]^ = 11646.2 ± 23.6) compared to normal polyclonal lambda (*m/z*^[2+]^ = 11428.1 ± 4.9). Conversely, patients with cardiac involvement exhibited a monoclonal lambda FLC with a “light” mass (*m/z*^[2+]^ = 11312.8 ± 16.1) relative to the normal control (Fig. 1e). This result must be interpreted with caution given the small sample size of this study, but we hope to confirm and explore the significance of this finding in a larger cohort of patients. In patients with kappa AL amyloidosis, whilst a monoclonal peak was apparent, no substantial difference in FLC molecular mass was observed when compared to normal sera (Fig. 1e).

This proof of concept study demonstrates the utility of a novel MALDI-TOF-MS technique to detect and characterise monoclonal FLC in AL amyloidosis. The Mayo group has led the way in MASS-FIX for detection of serum monoclonal immunoglobulins and FLCs, with sensitivities similar to current electrophoretic and nephelometric/turbidimetric methods^1^. The FLC-MS method described here substantially extends on previous findings by demonstrating i) high diagnostic sensitivity and specificity, in this small sample; 2) 100% concordance with immunohistochemistry results; and iii) crucially identifying monoclonal light chains in patients in serological CR but with persistent MRD.

In conclusion, the unique molecular location of FLC on MS can facilitate the detection of monoclonal amyloidogenic FLCs which may allow more accurate monitoring and more informed treatment decisions based on the detection of monoclonal pathogenic FLC component. The simplicity of this MALDI-TOF assay may allow for make it easier to implement in routine laboratories (if the findings are confirmed in larger studies) and the ability of MALDI-TOF MS to analyse intact FLCs may help in capturing crucial post-translational modifications, which may be key in the pathogenicity of FLC in AL amyloidosis. A study of a large cohort of uniformly treated patients with AL amyloidosis is in progress to confirm our findings and to assess the impact of FLC-MS on survival and organ response outcomes.
